# Genital infections and risk of premature rupture of membranes in Mulago Hospital, Uganda: a case control study

**DOI:** 10.1186/s13104-015-1545-6

**Published:** 2015-10-16

**Authors:** Sarah Nakubulwa, Dan K. Kaye, Freddie Bwanga, Nazarius Mbona Tumwesigye, Florence M. Mirembe

**Affiliations:** Department of Obstetrics and Gynecology, School of Medicine, Makerere University College of Health Sciences, P O Box 7072, Kampala, Uganda; Department of Microbiology, Makerere University College of Health Sciences, P O Box 7072, Kampala, Uganda; School of Public Health, Makerere University College of Health Sciences, P O Box 7072, Kampala, Uganda

**Keywords:** Genital infections, Premature rupture of membranes (PROM), Risk, Uganda

## Abstract

**Background:**

Inflammatory mediators that weaken and cause membrane rupture are released during the course of genital infections among pregnant women. We set out to determine the association of common genital infections (*Trichomonas vaginalis*, syphilis, *Neisseria gonorrhea, Chlamydia trachomatis*, Group B Streptococcus, Bacterial vaginosis, Herpes Simplex Virus Type 2 and candidiasis) and premature rupture of membranes in Mulago hospital, Uganda.

**Methods:**

We conducted an unmatched case–control study among women who were in the third trimester of pregnancy at New Mulago hospital, Uganda. The cases had PROM and the controls had intact membranes during latent phase of labour in the labour ward. We used interviewer-administered questionnaires to collect data on socio-demographic characteristics, obstetric and medical history. Laboratory tests were conducted to identify *T. vaginalis*, syphilis, *N. gonorrhea, C. trachomatis*, Group B Streptococcus, Bacterial vaginosis, Herpes Simplex Virus Type 2 (HSV-2) and candidiasis. Logistic regression models were used to estimate the odds ratios (OR) and 95 % CI of the association between genital infections and PROM.

**Results:**

There was an association between PROM and abnormal vaginal discharge (OR = 2.02, 95 % CI 1.10–3.70 and AOR = 2.30, 95 % CI 1.18–4.47), presence of candidiasis (OR = 0.27, 95 % CI 0.14–0.52 and AOR = 0.22, 95 % CI 0.10–0.46) and *T. vaginalis* (OR = 2.98, 95 % CI 1.18–7.56 and AOR = 4.22, 95 % CI 1.51–11.80). However, there was no association between PROM and presence of *C. trachomatis* (OR = 2.05, 95 % CI 0.37–11.49) and HSV-2 serostatus (OR = 1.15, 95 % CI 0.63–2.09). Few or no patients with Bacterial vaginosis, *Neisseria gonorrhoea*, Group B streptococcus or syphilis were identified among the cases and controls. Co-infection of Trichomoniasis and candidiasis was not associated with PROM (AOR = 1.34, 95 % CI 0.16–11.10). Co infection with *T. vaginalis* and *C. trachomatis* was associated with PROM (OR = 3.09, 95 % CI 1.21–7.84 and AOR = 4.22, 95 % CI 1.51–11.83).

**Conclusion:**

*Trichomonas vaginalis* alone, *T. vaginalis* with *C. trachomatis* co-infection and abnormal per vaginal discharge were found as risk factors for PROM. There was no association of HSV-2 serostatus, syphilis, *N. gonorrhea, C. trachomatis*, Group B Streptococcus and Bacterial vaginosis with PROM. Candidiasis seemed to have a protective effect on PROM.

## Background

Genital infections are implicated in significant morbidities among women of reproductive age group especially during the pregnancy period [1]. Inflammatory cells produced by genital infections are implicated in weakening of the fetal membranes among pregnant women thus causing premature rupture of membranes (PROM) [2]. PROM is defined as rupture of membranes before onset of labour [3, 4].

PROM may occur when the fetus is 37 weeks or more of gestation (term PROM) or before 37 weeks’ gestation (pre-PROM) [5]. PROM predisposes pregnant women and their unborn children to adverse outcomes such as maternal infection, fetal infection, umbilical cord compression and prolapse, fetal demise, low Apgar score, pulmonary hypoplasia, preterm delivery, low birth weights and fetal deformation [5]. Worldwide the prevalence of PROM ranges between 2 and 10 % [6]. In Mulago Hospital, 12 % of the admissions in the high risk labour ward have PROM as per the obstetrics and gynaecology departmental records of the year 2012.

The aetiology of PROM is diverse [7]. It includes polyhydramnios, cervical incompetence, uterine abnormality, trauma and previous cervical conization or cone biopsy [5, 7]. Other factors that have been implicated include past obstetric history of PROM, black race, smoking of cigarettes, poor nutrition and genital infections [5].

Genital infections [such as *Chlamydia trachomatis* (CT), *Trichomonas vaginalis* (TV), candidiasis, syphilis, bacterial vaginosis, *Neisseria gonorrhoea* and Group B Streptococcus] have been found to be associated with PROM [8]. *T. vaginalis* was found to weaken membranes in vitro studies and prospective studies have found an association between *T. vaginalis* and PROM [9, 10]. Studies by Chow and by Blas have demonstrated association of Chlamydial infections with occurrence of PROM [11, 12].

Women with Bacterial vaginosis BV (a condition where the normal flora of the vagina is disrupted causing abnormal vaginal discharge) compared with women without BV were more likely to develop PROM [8]. Maternal syphilis was also found associated with poor obstetric outcomes including prematurity; a common outcome of preterm PROM [13]. The association of candidiasis with PROM is still controversial but recent studies showed a positive association that is supported by the reduction in incidence of PROM through treatment of candidiasis [14]. Having *Neisseria gonorrhoea* (NG) during pregnancy conferred a 6 times higher risk of developing PROM compared to not having NG [15]. The evidence that Group B streptococci (GBS) may be a cause of PROM was supported by the fact that GBS causes an inflammatory responses in fetal membranes in experimental and epidemiological studies [3].

Genital HSV-2 is a frequent infection among women in child bearing age group globally [16]. There is experimental evidence that herpetic infection in the cervix causes release of cytokines and other inflammatory mediators [17]. These inflammatory mediators when released during infections of the genital tract of pregnant women are known to weaken and cause membrane rupture [18].

Despite the plausible association between genital infections and PROM, the findings from many studies are still inconsistent and it is unclear whether the proportion of women with confirmed genital infections differs among women with PROM and those without PROM. The objective of this study was to determine the association between common genital infections (*T. vaginalis*, Syphilis, *N. gonorrhea, C. trachomatis*, Group B Streptococcus, Bacterial vaginosis, candidiasis and HSV-2) and PROM among women attending delivering in the national referral hospital in Kampala, Uganda.

## Methods

### Study design and participants

An unmatched case–control study was conducted in the high risk labour ward in Mulago Hospital, Uganda between June and November 2013 to assess the association between PROM and genital infections. Mulago Hospital is one of the large hospitals in East Africa with 1500 bed capacity [19]. It serves as the national referral hospital for Uganda as well as the teaching hospital for Makerere University College of Health Sciences and functions as district Hospital for Kampala district. It is located approximately 3 km north of Kampala city center. The hospital has several departments including obstetrics and gynecology that offer specialized clinical care. The hospital has three labour wards namely; upper Mulago, lower Mulago (risk labour) and private (6D&E). The study was conducted at Mulago Hospital’s two general labour wards (upper and lower Mulago). The hospital has 23,000–30,000 deliveries a year and 12 percent of those had either term PROM or Pre-PROM. There are two antenatal clinics, one in Old Mulago and the other in New Mulago. The antenatal clinic in New Mulago Hospital is run 3 times a week and on average 150–200 patients are seen on each clinic day. All pregnant women in the antenatal clinic are offered HIV testing as part of prevention of mother and child transmission of HIV. The mothers have a code written on the file denoting whether positive or negative as per departmental records 2012.

For inclusion into the study, we selected participants presenting to the antenatal and labour wards at or above 28 weeks of gestation, 18 years and above who consented to the study. We recruited cases and controls by consecutive sampling procedure until the sample size was realized. Cases were women with confirmed premature rupture of membranes. The diagnosis of PROM was made from history of draining of clear fluid that wet the perineum and run along the thighs and legs plus sterile speculum examination. The diagnosis of PROM at speculum examination was made if there was observation of fluid pooling in posterior vaginal fornix or free flow of fluid from the cervix. The controls were women without PROM in latent phase of labour who were identified from the antenatal ward or the admission room of labour and met the inclusion criteria for controls. Women in latent phase of labour had uterine contractions, cervical effacement and dilatation up to 3 cm. The women in latent phase of labour with unruptured membranes were thus appropriate controls. These women were obviously not cases and could not be mistaken for such. In addition, there was ample time for consent. Assuming seroprevalence of genital HSV-2 of 49 % [20], 80 % study power, 95 % confidence interval and a minimum odds ratio of 2.4 of the association between PROM and genital infections, we estimated a minimum sample size of 87 cases and 87 controls (1:1) [21].

### Data collection

Interviewer-administered questionnaires were used to collect socio-demographic characteristics such as age, education, religion, occupation, tribe, gravidity and marital status. Sexual history such as age at first sexual experience, number of sexual partners, circumcision status of the partner and partner with multiple sexual partners was also collected. In addition, we collected gynaecological history [history of sexually transmitted infections in woman and partner(s), condom use in the woman and her partner(s) and history of operations on the cervix]. Other history sought included: obstetric history (past history of PROM and history of trauma to the cervix) and HIV status (from the antenatal and admission records. Other exposure variables were results from laboratory investigations for the selected genital infections (cervical swabs for *N. gonorrhea* and *C. trachomatis* and vaginal swabs for *T. vaginalis, Streptococcus agalactiae* and candidiasis,) and HSV-2 serology. Patients diagnosed with PROM were given prophylactic antibiotics whilst those with genital infections were given the appropriate medication.

### Assessment for genital infections

We drew two milliliters of blood from the vein in the cubital fossa using sterile technique and this was taken for HSV-2 and Syphilis serology. The blood samples were labelled and delivered to the Medical and molecular Laboratory of the Makerere university College of health sciences within 4 h. We performed a HerpeSelect 2 ELISA Ig G (to glycoprotein G) serology test (Focus Diagnostics, CA, USA). This test uses purified recombinant type-specific gG-2 antigen immobilized on polystyrene microwells and the procedure done was as per the manufacturer instructions. The results are reported as positive or negative (where index values <0.9 are negative and 1.1 and above are positive. Index values between 0.9 and 1.0 (inclusive) were classified as equivocal and these were then repeated. We reclassified OD index values from 1.1 to 3.4 as “low positive” and more than 3.4 as “high positive” [22].

We performed a RPR (Rapid Plasma Reagin) for syphilis using some of the sera above where results read reactive or non-reactive. Reactive test was demonstrated by agglomerates in the centre or the periphery of the test circle. A non-reactive test was indicated by an even appearance of the mixture. All reactive tests had a confirmatory ‘*Treponema pallidum* haemagglutination test (TPHA)’ performed [23].

We then removed five swabs (four vaginal and one cervical) for the other genital infections. Swab 1 was a cervical swab which was analysed by gram stain for *N. gonorrhoea* and by *Chlamydia antigen* test card for *C. trachomatis* [24].

Two high vaginal swabs were taken from the posterior vaginal fornix, one of them was taken for wet preparation and tested for candidiasis and *T. vaginalis* as described in Brown’s study [25]. The other high vaginal swab was taken for DNA PCR for *T. vaginalis* using the procedure used in a previous study [26]. The other two vaginal swabs were taken from the lateral vaginal walls. A gram stain for Bacterial vaginosis as well as culture and sensitivity for yeast cells were done from the vaginal swab three [27, 28]. Using vaginal swab 4, we performed culture and sensitivity tests for Group B Streptococcus [29].

### Quality control

We had training for all research assistants on research procedures including the consent process and data collection a week prior to the study and also had study meetings every week to review the challenges met and to resolve them. We also piloted the questionnaires. We used accredited laboratories that have buffer controls for all tests. The data was checked daily by the Principal Investigator for comprehensiveness prior to entry into the data base. Double data entry was done by two persons using the EPI-DATA programme and any missing data was checked against hospital records and other source documents.

### Data analysis

Data was analysed in STATA version 12 (Stata Corp., College Station, TX, USA). Categorical data for cases and controls was summarized as counts and proportions while continuous variables were summarized using means, and standard deviations. Some continuous variables like optical densities of HSV-2 were re-categorized into meaningful strata to be used for subsequent analysis. We initially compared the baseline characteristics of the cases and controls for comparability. To assess risk factors for PROM, we compared the presence of genital infections among the cases and controls using odds ratios and 95 % CIs and their p values. P values less than 0.05 were considered statistically significant. Multivariable analysis was done to determine the factors independently associated with PROM using logistic regression models. All variables with a p value of 0.05 or less and those with sound biologic plausibility were included in the regression models and adjusted odds ratios (AOR) with their 95 % Confidence intervals (CI) s were presented. The final regression model was evaluated using Hosmer–Lemeshow goodness-of-fit test [30].

### Ethical considerations

We obtained ethical approval from the Makerere University College of Health Sciences School of Medicine Research and Ethics Committee, and the Uganda National Council of Science and Technology. We obtained informed consent from all the participants and confidentiality was observed for all the study documents.

## Results

Distribution of age, gravidity, education level, marital status, occupation, and religion were similar among cases and controls (Table 1).

**Table 1:** Baseline characteristics of cases (PROM) & controls (NO PROM) in Mulago Hospital, Uganda

Variable	Cases (PROM) N = 87 (100 %)	Controls (No PROM) N = 87 (100 %)	P value
Religion			
Catholic	30 (36.0)	32 (37.0)	0.071
Muslim	27 (32.0)	20 (23.0)	
Protestant	24 (29.0)	12 (12.0)	
Born again	3 (4.0)	23 (26.0)	
Marital status			
Single	9 (10.0)	7 (8.0)	0.680
Married/cohabiting	76 (87.0)	66 (75.0)	
Divorce/separated	2 (3.0)	14 (17.0)	
Age			
<19.99	10 (11.4)	9 (10.3)	0.290
20–34.99	73 (84.0)	69 (79.4)	
≥35	4 (4.6)	9 (10.3)	
Gravidity			
Primegravida	31 (35.6)	31 (35.6)	1.000
Gravida 2–4	47 (54.1)	48 (55.2)	
Gravida 5 & above	9 (10.3)	8 (9.2)	
Education			
Primary and below	27 (31.0)	30 (34.0)	0.680
Secondary and tertiary	60 (69.0)	57 (66.0)	
Occupation			
House wife	31 (35.6)	33 (38.0)	0.200
Employed/business woman	34 (39.1)	33 (37.0)	
Others^a^	22 (25.3)	21 (25.0)	

The table shows that the distribution of age, gravidity, education level, marital status, occupation and religion of 87 cases and 87 controls was similar

^a^This includes those unemployed and students

There was an association between PROM and abnormal vaginal discharge (OR = 2.02, 95 % CI 1.10–3.70), presence of candidiasis (OR = 0.27, 95 % CI 0.14–0.52) and *T. vaginalis* (OR = 2.98, 95 % CI 1.18–7.56). There was an association between preterm gestation and PROM (OR = 4.27, 95 % CI 1.51–12.11). While there was an association between HSV-2 high positive optical densities and PROM (OR = 2.08, 95 % CI 1.07–4.07), there was no association between HSV-2 serostatus and PROM (56 versus 53 %, OR = 1.15, 95 % CI 0.63–2.09). However, there was no association between HIV serostatus and having PROM (OR = 0.99, 95 % CI 0.42–2.34). Similarly there was no association between presence of *C. trachomatis* and PROM (OR = 2.05, 95 % CI 0.37–11.49). Few or no patients with Bacterial vaginosis, *N. gonorrhoea*, Group B Streptococcus or syphilis were identified among the cases and controls (Table 2).

**Table 2:** Bivariate analysis for genital symptoms and infections among cases (had PROM) and controls (without PROM)

Variable	Cases N = 87 (100 %)	Controls N = 87 (100 %)	P value	OR (95 % CI)
Abnormal vaginal discharge				
Yes	47 (54.0)	32 (36.8)	*0.023*	*2.02 (1.10–3.70)*
No	40 (46.0)	55 (63.2)		
Painful genital ulcer history				
Yes	9 (10.3)	3 (3.4)	0.087	3.23 (0.84–12.37)
No	78 (89.7)	84 (56.6)		
HSV-2 serology				
Positive	49 (56.0)	46 (53.0)	0.648	1.15 (0.63–2.09)
Negative	38 (44.0)	41 (56.6)		
HSV-2 ELISA optical densities				
>3.4 (high positives)	32 (37.0)	19 (22.0)	*0.032*	*2.08 (1.07–4.07)*
≤3.4 (negative or low positive)	55 (63.0)	68 (78.0)		
HIV status				
Positive	12 (13.8)	12 (13.8)	0.098	0.99 (0.42–2.34)
Negative	75 (86.2)	75 (86.2)		
Candida				
Positive	18 (20.7)	43 (49.4)	*<0.001*	*0.27 (0.14–0.52)*
Negative	69 (79.3)	44 (50.6)		
*Trichomonas vaginalis*				
Positive	18 (20.7)	7 (8.0)	*0.02*	*2.98 (1.18–7.56)*
Negative	69 (79.3)	80 (92.0)		
Bacterial vaginosis				
Positive	0 (0.0)	2 (2.3)	^a^	
Negative	87 (100.0)	85 (97.7)		
*Neisseria gonococcus*				
Positive	0 (0.0)	1 (1.1)	^a^	
Negative	87 (100.0)	86 (98.9)		
*Chlamydia trachomatis*				
Positive	4 (4.6)	2 (2.3)	0.415	2.05 (0.37–11.49)
Negative	83 (95.4)	85 (97.7)		
Syphilis				
Positive	0 (0.0)	0 (0.0)	^a^	
Negative	100 (100.0)	100 (100.0)		

We assessed the association between PROM and: abnormal vaginal discharge, history of painful genital ulcer, candidiasis, *Trichomonas vaginalis,* preterm labour, HSV-2 serostatus, HSV-2 titres, HIV, bacterial vaginosis, *Neisseria gonorrhoea*, Group B streptococcus and with syphilis. The italicized odds ratios had a significant association with PROM (abnormal vaginal discharge, candidiasis, *Trichomonas vaginalis,* and preterm labour and HSV-2 titres)

^a^Means no further analysis was done for these variables due to few numbers

The independent risk factors for PROM at multivariable analysis were abnormal vaginal discharge (AOR = 2.30, 95 % CI 1.18–4.47) and *T. vaginalis* (AOR = 4.22, 95 % CI 1.51–11.80) as shown in model 1 (Table 3). Candidiasis was found to be protective for PROM (AOR = 0.22, 95 % CI 0.10–0.46).

**Table 3:** Genital infections and risk for PROM in Mulago Hospital

Variable	Unadjusted odds ratio (95 % CI)	Adjusted odds ratio (95 %) in model 1	Adjusted odds ratio (95 %) in model 2^a^	Adjusted odds ratio (95 %) in model 3^b^
Abnormal vaginal discharge				
Yes	*2.02 (1.10–3.70)*	*2.30 (1.18–4.47)*	*2.30 (1.18–4.40)*	*2.30 (1.18–4.46)*
No				
HIV status				
Positive	0.99 (0.42–2.34)	1.38 (0.51–3.75)	1.30 (0.51–3.72)	1.38 (0.51–3.75)
Negative				
*Trichomonas vaginalis*				
Positive	*2.98 (1.18–7.56)*	*4.22 (1.51–11.80)*	3.70 (0.92–14.90)	
Negative				
*Chlamydia trachomatis*				
Positive	2.05 (0.37–11.49)	1.91 (0.30–12.22)	1.92 (0.30–12.20)	
Negative				
Candida				
Positive	*0.27 (0.14–0.52)*	*0.22 (0.10–0.46)*	0.16 (0.02–1.22)	*0.22 (1.51–11.83)*
Negative				
HSV-2				
Positive	1.15 (0.63–2.09)	0.95 (0.48–1.88)	0.94 (0.48–1.87)	0.95 (0.48–1.88)
Negative				
Trichomonas and candida	*0.49 (0.27–0.90)*		1.34 (0.16–11.10)	1.34 (0.16–11.10)
Trichomonas and Chlamydia	*3.09 (1.21–7.84)*			*4.22 (1.51–11.83)*

In this table we have three models after multivariable analysis. Model 1 included abnormal vaginal discharge, HIV status *Trichomonas vaginalis,* *Chlamydia trachomatis,* candidiasis and HSV-2 serostatus. The italicized odds ratios were either independent risk factors for PROM (abnormal vaginal discharge and *Trichomonas*) or protective factors (candidiasis). Model 2 is included to assess whether co-infection with Trichomoniasis and candidiasis was a risk factor for premature rupture of membranes and found that this interaction was not a risk factor for PROM at multivariable analysis. Model 3 is included to assess whether co-infection with *Trichomonas vaginalis* and *Chlamydia trachomatis* was associated with PROM and it is shown that this interaction is a risk factor for PROM

^a^The model 2 assesses interactions between *Trichomonas vaginalis* and candidiasis

^b^The model 3 assesses interaction between *Trichomonas vaginalis* and *Chlamydia trachomatis*

Co-infection of Trichomoniasis and candidiasis was associated with PROM (OR = 0.49, 95 % CI 0.27–0.90) at bivariate analysis but not at multivariable analysis (OR = 1.34, 95 % CI 0.16–11.10) from model 2. From model 2, the only factor that had an independent association with PROM was abnormal per vaginal discharge (AOR = 2.30, 95 % CI 1.18–4.40) (Table 3).

Co infection with *T. vaginalis* and *C. trachomatis* was associated with PROM (OR = 3.09, 95 % CI 1.21–7.84 and AOR = 4.22, 95 % CI 1.51–11.84) as shown in model 3 (Table 3).

## Discussion

This study determined the association of selected genital infections (*T. vaginalis*, Syphilis, *N. gonorrhea, C. trachomatis*, group B Streptococcus, Bacterial vaginosis, candidiasis and HSV-2) and PROM in Mulago hospital, Uganda. We found out that the odds of association between PROM with abnormal vaginal discharge and *T. vaginalis* was twice and thrice that without PROM respectively. There was no association between HSV-2 serostatus, being HIV seropositivity or with presence of *C. trachomatis* with PROM. The association of HSV-2 and PROM is not explored in this paper. Our study found few or no patients with bacterial vaginosis, *N. gonorrhoea*, Group B streptococcus or syphilis were identified among the cases and controls. Candidiasis was found to be protective for PROM; patients with PROM were 73 % less likely to have candidiasis compared with those without PROM in this study.

The mechanisms of how infections increase the risk of PROM are through release of inflammatory cytokines and proteases [2]. There is epidemiological evidence by Maymon et al. [18] that inflammatory cytokines were increased in patients with PROM. Indeed, genital infection have been found as a risk of poor pregnancy outcomes PROM [31, 32]. Furthermore, women with herpetic infections had increased cytokines in the cervical cells [17]. In having some genital infections may impair antimicrobial role of the pregnant cervix making it more susceptible to other microbes [17].

The strong association between having *Trichomonas vaginalis* (TV) infection and PROM found in our study is supported by in vitro studies that demonstrated 80 % reduction in term fetal membranes tension leading to membrane rupture when incubated with fresh *T. vaginalis* isolates [9]. There is experimental evidence that inflammatory proteases are involved in the *T. vaginalis* induced membrane rupture [9]. Our results are also similar to results from a prospective study in Kashan (Iran) among 450 pregnant women where TV was associated with poor pregnancy outcomes such as preterm labour and PROM [33]. Similarly, epidemiological evidence from the USA showed TV was also associated with PROM [10]. In the US population overall TV prevalence was 12 % among blacks which is slightly lower though comparable to the proportion in our study which was 14 % overall and 20.7 % among the women with PROM. Contrary to our findings, an analysis of secondary data for 428 women in South Africa concluded that treatment of *T. vaginalis* in pregnant women did not reduce incidence of PROM. However, this was a smaller study compared to the previous large US studies and more prospective interventional studies towards *T. vaginalis* are still needed in low resource settings [34]. *T. vaginalis* is thus a risk factor for PROM.

The positive association between having an abnormal vaginal during pregnancy and PROM is also supported by studies done by Kaye et al. [35] in Uganda and Karat et al. [36] in India. The association between PROM and abnormal discharge, a common symptom of genital infections is most likely via inflammatory mediators [3]. One study that found no association between abnormal vaginal discharge and PROM was done in two centers in Brazil where women with genital infections had had full treatment courses, a factor that was not assessed in our study [37]. It was however not possible, from our findings to match each specific vaginal discharge to the confirmed genital infections as this was not differentiated from our study.

The association between candidiasis and rupture of membranes is still unclear. There is evidence of release of inflammatory cytokines during candida infestation [38]. We had postulated that these cytokines would cause membrane rupture. However our study and in a case–control study in India, patients with PROM were less likely to have candidiasis compared to those without PROM [36]. There is a possibility that the liquor amnii in patients with PROM washed out the yeast cells leading to non-detection but this is not proven. In contrast, a study by Rasti et al. [33] did not find candida as a risk factor for PROM. Furthermore, there was indirect evidence that candidiasis increased risk of membrane rupture in a study where use of antifungal medication reduced preterm labour and PROM [14]. The association of candida with PROM is still currently non- conclusive and needs to be explored more.

Our study showed no significant association between PROM and *Chlamydiatrachomatis* (CT) yet studies by Chow et al. [11] in California and Blas et al. [12] in Washington have demonstrated association between CT and PROM. *C.trachomatis* infection in pregnancy women is known to cause release of inflammatory mediators that are implicated in membrane rupture [4]. In our study population, this relationship between *C.trachomatis* and PROM may not have been demonstrated because only 4.6 % (4/87) of the cases and 23 % (2/87) had with confirmed CT which was lower than the 13 % Chlamydial case reports in Chow’s study. The cohort by Blas was a large prospective study and was also able to demonstrate an association of *C. trachomatis* with PROM. In our study we were not able to demonstrate with evidence that *C. trachomatis* is a risk factor for PROM.

Our study showed no significant association between HIV sero- status and PROM. However in a prospective cohort of 68 women with advanced HIV and 68 HIV negative women showed an association between HIV and PROM (31 and 9 % respectively, p < 0.001) by Musana et al. [39]. The explanation of the relationship between HIV and PROM is via inflammatory poly morphonuclear cells that have been shown to be increased among HIV positive women cervical fluid [40]. Our study did not demonstrate this because we did not stage the patients according to their WHO clinical staging of the HIV disease. Musana’s study enrolled patients who had their WHO clinical staging done and demonstrated that PROM was associated with advanced HIV/AIDS disease stage.

None of the patients with PROM and 2.3 % of those without PROM in our study had bacterial vaginosis. In one study where an association of Bacterial vaginosis was found with PROM 29 % of patients with PROM and 11 % of patients without PROM had Bacterial vaginosis [41]. None of the patients with PROM and 1.1 % of those without PROM in our study had *N. gonorrhoea.* In the study by Donders,among pregnant women in south Africa, where an association was found between PROM and gonococcal infections, the prevalence of *N. gonorrhoea* was there was 4.5 % in the population studied (6 times more among patients with PROM compared to those without PROM) [15]. In our study we did not identify any patient with GBS. However, Kessous found an association of PROM with GBS, where the prevalence of GBS in the women with PROM was 10.7 % while that among those without PROM was 7.9 % [42]. There is evidence that Group B Streptococcus, *N. gonorrhoea* and bacterial vaginosis cause release of cytokines and other inflammatory modulators that can cause membrane rupture [4].

We did not identify any patient with syphilis yet in the Tanzanian study where an association between PROM and syphilis was found, the prevalence of syphilis was 8 % [13]. Treponema Pallidum, the bacteria responsible for syphilis is also known to cause release of cell lysing enzymes when it infects the genital mucosa [43]. However, our study population had very low prevalence of GBS, *N. gonorrhoea*, bacterial vaginosis and syphilis thus no inferences could be drawn. The definitive reasons for the low prevalence of some genital infections remain unclear but could be that the patients may have been on antibiotics such as erythromycin which is given to all women with PROM. *N. gonorrhea*, syphilis and GBS are susceptible to erythromycin. We thus do not have evidence that Group B Streptococcus, *N. gonorrhoea*, Bacterial vaginosis and syphilis are risk factors for PROM in our setting.

### Co-infections

Co-infection with *T. vaginalis* and *C. trachomatis* was a risk factor for premature rupture of membranes in our study. Experimental studies have indeed shown that infections among patients presenting with premature rupture of membranes are multi microbial [44]. French et al. (2006) found that single infection such as *T. vaginalis* or *C. trachomatis* and those with multiple infections including *T. vaginalis* –*C. trachomatis* co-infections were risk factors for PROM [45]. The possible explanation for increased risk of PROM in multiple infections may be due to additive inflammatory response as explained by a model involving mixed bacterial infections [44].

### Limitations

We did not collect the data of history of drug use and type of drug used at onset of PROM. Due to the low prevalence of *N. gonorrhoea*, syphilis, Group B Streptococcus, bacterial vaginosis and *C. trachomatis*, we need a larger study to assess these infections and risk of PROM.

### Implications

We need implementation research to strengthen routine screening and management for *T. vaginalis* among pregnant women so as to assess the effect of the intervention on reduction of incidence of PROM. We propose to clinicians and programme managers that they need to conduct further tests to look for other infections in patients with one infection, so as to optimize prevention and care in women with premature rupture of membranes. Further research to assess the effect of candida colonization on occurrence of PROM is also imperative. A prospective study on the membranes and placentae in patients with PROM found to have Trichomoniasis is needed to define the causative pathway further. We also need a larger study to assess *C. trachomatis*, *N. gonorrhoea*, GBS, syphilis and Bacterial vaginosis for risk of PROM.

## Conclusions

*Trichomonas Vaginalis* alone, *T. vaginalis* with *C. trachomatis* co-infection and abnormal per vaginal discharge were found as a risk factors for PROM in Mulago hospital, Uganda. There was no association of HSV-2 serostatus, Syphilis, *N. gonorrhea, C. trachomatis*, Group B streptococcus and Bacterial vaginosis with PROM. Candidiasis seemed to have a protective effect on PROM.

## Abbreviations

HSV-2: herpes simplex virus type-2
PROM: premature rupture of membranes
CT: *Chlamydia trachomatis*
HIV: human immunodeficiency virus
TV: *Trichomonas vaginalis*
TPHA test: *Treponema pallidum* haemagglutination test
PVD: per vaginal discharge
GBS: Group B Streptococcus
